# Comparative efficacy of conventional and nano-formulations of silica and chitosan against *Fusarium oxysporum* causing wilt in pea (*Pisum sativum*)

**DOI:** 10.1186/s12870-026-08334-y

**Published:** 2026-03-07

**Authors:** Hany H. A. El-Sharkawy, Mohamed S. Abbas, Amira S. Soliman, Seham A. Ibrahim, Ibrahim A. I. El-Nady, Doaa A. Galilah

**Affiliations:** 1 Mycology Research and Diseases Survey Department, Plant Pathology Research Institute, Agricultural Research Center, Giza, Egypt; 2 Natural Resources Department, Faculty of African Postgraduate Studies, Cairo University, Giza, Egypt; 3 Department of Agricultural Botany, Faculty of Agriculture, Zagazig University, Zagazig, Egypt; 4 Botany Department, Faculty of Science, Mansoura University, Mansoura, Egypt

**Keywords:** Pea, *Fusarium oxysporum* f. sp. *pisi*, Silicon, Chitosan, Nanoparticles, Plant defense, Eco-friendly disease management

## Abstract

**Background:**

Vascular wilt caused by *F. oxysporum* f. sp. *pisi* is among the most destructive soil-borne diseases threatening pea cultivation worldwide, particularly under warm and humid conditions that favor pathogen proliferation. Conventional management strategies rely heavily on chemical fungicides; however, their extensive use has resulted in environmental contamination, health concerns, and the emergence of resistant pathogen strains. Recently, nanotechnology-based disease management has emerged as a sustainable alternative. Silicon (Si) and chitosan (Cs) are recognized as effective resistance inducers, and their nano-formulations (Si-NPs and Cs-NPs) may further enhance disease suppression and activation of plant defense responses. An integrated strategy combining in vitro antifungal assays, greenhouse disease evaluation, and multi-level physiological and anatomical analyses was employed. Nevertheless, comparative evaluations of these nano-elicitors against *F. oxysporum* in pea remain limited, particularly under greenhouse conditions.

**Results:**

In vitro assays demonstrated that Si-NPs and Cs-NPs completely inhibited mycelial growth (100% inhibition) of *F. oxysporum* f. sp. *pisi* at 150 µg/mL, whereas conventional Si (8 g/L) achieved only 50.7% inhibition, and Cs (8 g/L) achieved 100% inhibition. Under greenhouse conditions, seed treatment with Si-NPs markedly reduced the severity of Fusarium wilt in pea plants by 72.7%, showing a level of protection comparable to the systemic fungicide control. Likewise, Cs-NPs significantly decreased wilt severity by 72.2%, whereas conventional Si and Cs treatments were less effective, achieving 60.6% and 63.6% reductions, respectively. Biochemical analyses showed that Si-NPs increased total phenols by 116.8%, polyphenol oxidase (PPO) by 102%, and peroxidase (POD) by 31.4%, followed by Cs-NPs (106.6%, 72.1%, and 27.9%, respectively). Si-NPs also enhanced chlorophyll a, b, total chlorophyll, and carotenoids by 108.9%, 43.7%, 111.7%, and 69.2%, while reducing membrane permeability and lipid peroxidation by 37.1% and 69.8%, respectively. Si-NPs increased total root section thickness, cortex thickness, vascular cylinder thickness, and xylem vessel diameter by 15.81%, 12.51%, 21.00%, and 90.60%, respectively, while Cs-NPs enhanced these traits by 13.73%, 7.85%, 19.02%, and 63.76%. Notably, nano-formulations—particularly Si-NPs—promoted coordinated reinforcement of root tissues, reflected by optimized cortex-to-vascular cylinder ratios, which likely contributed to enhanced resistance against *F. oxysporum* without impairing vascular function.

**Conclusions:**

The findings of this study demonstrate that Si-NPs and Cs-NPs provide superior protection against *F. oxysporum*-induced wilt in pea plants compared to their conventional counterparts. Their enhanced performance is attributed to a dual mode of action—direct antifungal activity and the stimulation of plant defense responses. These results underscore the potential of Si-NPs and Cs-NPs as promising, eco-sustainable alternatives to synthetic fungicides for integrated disease management in legumes. Future studies should focus on evaluating their long-term field efficacy, formulation stability, and environmental safety to facilitate large-scale agricultural applications.

**Supplementary Information:**

The online version contains supplementary material available at 10.1186/s12870-026-08334-y.

## Background

Pea (*Pisum sativum* L.) is one of the most important legume crops globally, valued for its high nutritional content, protein-rich seeds, nitrogen-fixing ability, which contributes to soil fertility and sustainable agricultural systems [1–3]. Despite its importance, pea production is severely constrained by soil-borne fungal pathogens, among which *F. oxysporum* f. sp. *pisi* is particularly devastating [4–6]. *F. oxysporum* f. sp. *pisi* causes vascular wilt, leading to severe yield losses through disruption of water transport, wilting, and eventual plant death [7–9]. The persistence of *F. oxysporum* chlamydospores in soil for extended periods makes disease management particularly challenging [7, 10].

Conventional management strategies, including chemical fungicides and resistant cultivars, have shown limited and often transient success. Fungicides, though initially effective, pose environmental and health hazards and accelerate the emergence of resistant fungal strains in several crops [11–13]. Similarly, host plant resistance is often short-lived due to the genetic variability and adaptability of *F. oxysporum* populations [14, 15]. The limitations of fungicides and resistance breeding highlight the need for sustainable, eco-friendly alternatives for controlling Fusarium wilt in pea [14, 16–18].

Natural defense inducers such as silicon (Si) and chitosan (Chit) have emerged as promising environmentally friendly strategies. Silicon reinforces plant cell walls, improves lignification, and modulates signaling pathways associated with systemic acquired resistance (SAR) and induced systemic resistance (ISR), enhancing tolerance against vascular pathogens [12, 16, 19, 20]. Chitosan, a deacetylated derivative of chitin, elicits plant immunity by inducing reactive oxygen species (ROS), pathogenesis-related (PR) proteins, and phenolic compounds while also exerting direct antifungal effects via membrane disruption and inhibition of spore germination. These compounds can also mitigate soil stresses and promote plant growth and defense, suggesting their potential for integrated disease management [19, 21].

Nanotechnology has further enhanced the efficacy of these agents. Nano-formulations such as silicon nanoparticles (Si-NPs) and chitosan nanoparticles (Chit-NPs) provide superior reactivity, controlled release, and enhanced tissue permeability, allowing more efficient interaction with host plants and pathogens [14, 22]. The high surface-area-to-volume ratio of nanoparticles strengthens defense elicitation and improves antifungal activity compared to bulk forms. Recent studies highlight the dual benefits of nanoparticle-based approaches: promoting plant growth and photosynthetic potential while suppressing soil-borne pathogens [22, 23]. For instance, combined application of culture filtrates with nanoparticles has been shown to alleviate soil stresses, suppress pathogens, and improve yield in grapes, onion and tomato [14, 17, 18]. Nano- or elicitor-mediated defense induction has also been applied successfully against *Plasmopara viticola*, *Botrytis cinerea* and *F. graminearum*, demonstrating broad-spectrum potential [6, 14, 15]. Despite these advances, comparative studies evaluating conventional versus nano-formulations of Si and Cs against *F. oxysporum* in pea are limited. Assessing the relative efficacy of bulk and nano-formulations in activating host defenses, suppressing pathogen growth, and improving field-level disease management is critical for developing sustainable and environmentally friendly strategies.

Therefore, this study aimed to evaluate the antifungal efficacy of conventional and nano-formulations of Si and Cs against *F. oxysporum* f. sp. p*isi* in pea plants. The study integrated in vitro antifungal assays, greenhouse experiments, and comprehensive biochemical and physiological analyses to elucidate the mechanisms associated with disease suppression and induced resistance. In addition, the effects of these treatments on plant growth, yield-related traits, and seed quality under pathogen stress were assessed.

## Methods

### Pathogen isolation and verification

Pea plants showing typical wilt symptoms were collected from major pea-growing areas in Egypt, including multiple fields in the Sharqia and Dakahlia Governorates. Infected root and stem samples were surface-sterilized and used for isolation of the causal pathogen on potato dextrose agar (PDA). The purified fungal isolates were identified based on cultural and microscopic characteristics according to the taxonomic keys of Booth [24], confirming *F. oxysporum* as the primary pathogen. The selected isolate was previously tested for pathogenicity through preliminary inoculation assays, confirming its ability to induce typical wilt symptoms in pea. During the current experiment, the pathogen was re-isolated from symptomatic plants to fulfill Koch’s postulates, ensuring that observed symptoms were caused by the target pathogen.

### Plant material

Certified seeds of pea cultivar Master-B were obtained from the Horticulture Research Institute, Agricultural Research Center (ARC), Giza, Egypt. Seeds were surface-sterilized with 1% sodium hypochlorite for 2 min, rinsed thoroughly with sterile distilled water, and used for seed treatment experiments as described below.

### Chemical inducers and fungicide

Bulk Si and Cs were purchased from El Nasr Company for Chemicals and Pharmaceuticals (Cairo, Egypt). Solutions were prepared at concentrations of 4, 6, and 8 g L⁻¹ for conventional treatments. The systemic fungicide Topsin-M 70% WP (Nippon Soda Co., Japan), containing thiophanate-methyl, was used as a positive control at a rate of 3 g kg⁻¹ of seeds.

### Nanoparticles characterization

#### Characterization of Si-NPs

Si-NPs used in the present study were obtained from Nano Gate (Cairo, Egypt) as a white powder with a molecular weight of 60.08 g mol⁻¹. According to the manufacturer’s technical specifications, the nanoparticles were synthesized using a wet-gel silica slurry method through the drop-wise addition of hydrochloric acid into an aqueous sodium silicate solution. The morphology and structural characteristics of the Si-NPs were evaluated using transmission electron microscopy (TEM) operated at an accelerating voltage of 200 kV (JEOL JEM-2100, Japan), which confirmed a predominantly spherical-like morphology with an average particle size of 20 ± 4 nm. The crystalline structure was examined by X-ray diffraction (XRD) analysis using a copper (Cu) radiation source, revealing a characteristic broad diffraction peak indicative of the amorphous nature of the prepared silica nanoparticles. Detailed TEM micrographs, XRD patterns, and Fourier-transform infrared (FTIR) spectra are provided in Supplementary Figure S1.

#### Characterization of CsNPs

CsNPs were provided by the Nanotechnology and Advanced Nano-Materials Laboratory, Plant Pathology Research Institute, ARC, Egypt. The CsNPs were prepared as a liquid solution with a concentration of 1 g/L using low molecular weight chitosan. The particle size distribution was determined using Dynamic Light Scattering (DLS) with a Nanosizer Nano ZS instrument (Malvern Instruments, UK), which revealed an average particle size of 15 ± 3 nm. Additionally, the crystalline structure of the synthesized CsNPs was confirmed through X-ray Diffraction (XRD) pattern analysis. The corresponding DLS distribution curves and XRD patterns are provided in Supplementary Figure S2.

This level of characterization was considered sufficient to ensure reproducibility and to support the biological interpretations presented in this study.

### Reagents for biochemical and physiological analyses

All analytical-grade reagents were obtained from Sigma-Aldrich (USA), including:

- Catechol for PPO assays
- Pyrogallol for POD assays
- 0.1 M phosphate buffer (pH 7.2) for enzyme extraction
- Ethanol and methanol for pigment extraction
- Folin–Ciocalteu reagent for total phenolics determination
- Thiobarbituric acid (TBA) for malondialdehyde estimation
- Sodium carbonate for pigment quantification

### In vitro antifungal activity of bulk and nano-scale formulations of Si and Cs against *F. oxysporum*

The in vitro antifungal activity of Si and Cs, in both their conventional and nano-formulations, against *F. oxysporum* was assessed using the poisoned food technique as described by Rashad et al. [25]. The evaluated treatments included conventional Si and Cs, as well as their corresponding nano-formulations (Si-NPs and Cs-NPs). Conventional Si and Cs were tested at concentrations of 4, 6, and 8 g L⁻¹, whereas Si-NPs and Cs-NPs were applied at concentrations of 50, 100, and 150 µg mL⁻¹. Control plates containing unamended PDA served as the untreated control.

For each treatment, a 5-mm-diameter mycelial disc obtained from the margin of an actively growing *F. oxysporum* culture was centrally placed on PDA medium amended with the respective concentrations of the tested inducers in 9-cm Petri dishes. The plates were incubated at 27 ± 1 °C for 7 days. Each treatment was replicated four times, and the experiment was conducted twice to ensure reproducibility. Radial fungal growth was measured when the mycelium in the control plates reached the edge of the Petri dishes. The percentage of mycelial growth inhibition was calculated according to the following formula Rashad et al. [26]:

Inhibition (%) = [(R₁ - R₂) / R₁] × 100

where R₁ represents the mean colony diameter (mm) in the control plates and R₂ represents that in the treated plates.

### Application method of chemical inducers and pathogen inoculation

Healthy pea seeds (cv. Master-B) were surface-sterilized with 1% sodium hypochlorite for 2 min, thoroughly rinsed with sterile distilled water, and air-dried under sterile conditions. Seeds were subjected to a single pre-sowing treatment by soaking in the respective chemical inducer solutions for 3 h at room temperature. Si and Cs were applied at concentrations of 4, 6, and 8 g L⁻¹, whereas their nano-formulations (Si-NPs and Cs-NPs) were applied at 150 µg mL⁻¹, previously determined as the minimum inhibitory concentration (MIC) in vitro. After treatment, seeds were air-dried and immediately sown.

The experiment was conducted under greenhouse conditions (25 ± 2 °C) using a randomized experimental design. Sterilized plastic pots (25 cm diameter) were filled with 10 kg of sandy clay loam soil artificially infested with *F. oxysporum* at a rate of 3% (w/w), following El-Sharkawy [27]. Soil infestation was performed 10 days prior to sowing to ensure uniform pathogen establishment. Ten treated seeds were planted per pot, and each treatment was replicated six times.

Two control treatments were included: a negative control (uninfested, untreated plants) and a positive control (untreated plants grown in infested soil). Additional treatments included the fungicide Topsin-M 70 WP (3 g L⁻¹), conventional Si and Cs (4, 6, and 8 g L⁻¹), and their nano-formulations (Si-NPs and Cs-NPs) at 150 µg mL⁻¹. No additional foliar spray or soil drench applications were applied during the experimental period.

### Assessment of fusarium wilt severity

At 45 days post-planting, five plants per treatment were randomly selected and evaluated for Fusarium wilt disease severity (DS%) following the six-grade disease rating scale proposed by Bani et al. [28]. The scale was defined as follows:

0 = no visible symptoms;

1 = chlorosis or wilting of one basal leaf, pale yellow-green color, and slight downward curling of leaf margins and stipules;

2 = chlorosis or wilting of a few basal leaves without visible stunting;

3 = chlorosis or wilting of several basal leaves accompanied by mild stunting and generalized leaf yellowing;

4 = chlorosis or wilting of most leaves, severe stunting, and drying of the lower foliage;

5 = complete wilting and death of the plant.

The DS% was calculated using the following equation:

DS (%)=∑(a×b)/A×K×100.

where:

*a* = number of plants within each disease category,

*b* = corresponding severity rating,

*A* = total number of assessed plants, and.

*K* = highest severity level.

### Biochemical and physiological analyses

Biochemical and physiological assessments were carried out on pea plants at different growth stages to evaluate the influence of Si and Cs, in both conventional and nano forms, on defense-related responses and physiological performance under *F. oxysporum* infection. Photosynthetic pigments were quantified from fresh leaf tissues sampled 40 days after sowing (DAS). Pigments were extracted for 24 h in methanol containing traces of sodium carbonate, and their absorbance was measured spectrophotometrically according to the method of Lichtenthaler and Wellburn [29].

The activities of PPO and POD enzymes were determined from fresh root tissues sampled 15 DAS. PPO activity was assayed following Maria [30], while POD activity was determined according to Maxwell and Bateman [31]. Total phenolic content was measured 15 DAS using the Folin–Ciocalteu reagent, as described by Maliak and Singh [32], and expressed as mg gallic acid equivalent (GAE) per gram fresh weight. Lipid peroxidation, expressed as malondialdehyde (MDA) content (µmol g⁻¹ fresh weight), was quantified based on the TBA reaction according to Shao et al. [33], using an extinction coefficient of 155 mM⁻¹ cm⁻¹. Samples were collected 60 days after sowing.

Membrane permeability (MP%) was determined 50 DAS by measuring the electrolyte leakage percentage (ELP) using a conductivity meter (EC meter; Hanna Instruments, UK), following the method described by Farouk et al. [6]. MP% was calculated as (EC₁/EC₂) × 100, where EC₁ and EC₂ represent the initial and final electrical conductivity readings, respectively. Seed quality parameters were determined in fresh pea seeds harvested at maturity.

Seed quality parameters were evaluated using freshly harvested pea seeds collected 70 DAS. Total soluble solids (TSS) were determined in fresh seed extracts using a hand-held refractometer. Total carbohydrate content was quantified according to the method described by Dubois et al. [34], while protein content was estimated following Yenu and Follard [35]. The use of freshly harvested seeds at this developmental stage enabled an accurate assessment of the physiological status and nutritional quality of the crop at harvest.

### Root anatomical analysis

Root anatomical alterations were examined 40 days after sowing to evaluate the structural responses of pea plants to nanoparticle treatments. Samples were collected from plants treated with Cs-NPs and Si-NPs at a concentration of 150 µg mL⁻¹. Root segments (approximately 1 cm in length) were excised from the maturation zone, fixed in formalin–acetic acid–alcohol (FAA) solution, dehydrated through a graded ethanol series, and embedded in paraffin wax. Transverse Sects. (10–12 μm thick) were prepared and stained with safranin and fast green, following the standard microtechnique described by El-Sharkawy et al. [27].

Microscopic observations were conducted using a light microscope (Leica DM500, Germany), and quantitative measurements were recorded using an ocular micrometer. The evaluated anatomical parameters included total root section thickness (µm), cortex thickness (µm), vascular cylinder thickness (µm), and xylem vessel diameter (µm). Percentage changes in each anatomical trait relative to the infected untreated control were calculated using the formula:

$$\pm\%=\left(\mathrm{Control}-\mathrm{Treatment}\right)\;/\;\mathrm{Control}\;\times\;100$$

In addition, the cortex thickness-to-vascular cylinder thickness ratio was calculated and expressed as a percentage to provide a size-independent indicator of tissue allocation and structural modification. These anatomical measurements were used to assess the extent of root tissue reinforcement and vascular adjustment associated with enhanced structural resistance induced by the nano-formulations.

### Vegetative growth parameters

To assess the effect of nano-priming treatments on the vegetative growth of pea plants under *F. oxysporum* stress, ten plants were randomly selected from each treatment at 45 days post-inoculation. Plants were carefully uprooted and thoroughly washed under running tap water to remove adhering soil particles. The following growth parameters were measured: shoot height (cm), root length (cm), and leaf area (cm²). Fresh weights of shoots and roots were recorded immediately after harvest, while dry weights were determined after oven-drying the samples at 80 °C until a constant weight was achieved. These parameters were used to evaluate plant physiological vigor and biomass accumulation during the active vegetative growth stage.

### Yield and its components

At physiological maturity, ten plants were randomly selected from each treatment and harvested to evaluate the effects of the applied inducers on crop productivity. Yield parameters assessed included pod length (cm), pod diameter (cm), number of pods per plant, and 100-seed weight (g). All yield components were recorded after air-drying the samples under uniform environmental conditions to standardize moisture content and ensure measurement accuracy. 

### Statistical analysis

All experimental data were statistically analyzed using the CoStat Software package (version 6.4; CoHort Software, Monterey, CA, USA) following the procedures described by CoStat [36]. Analysis of variance (ANOVA) was performed to evaluate the significance of differences among treatments. Mean separation was conducted using Duncan’s multiple range test [37] at a probability level of *P* ≤ 0.05.

### Vegetative growth parameters

To assess the effect of nano-priming treatments on the vegetative growth of pea plants under *F. oxysporum* stress, ten plants were randomly selected from each treatment at 45 days post-inoculation. Plants were carefully uprooted and thoroughly washed under running tap water to remove adhering soil particles. The following growth parameters were measured: shoot height (cm), root length (cm), and leaf area (cm²). Fresh weights of shoots and roots were recorded immediately after harvest, while dry weights were determined after oven-drying the samples at 80 °C until a constant weight was achieved. These parameters were used to evaluate plant physiological vigor and biomass accumulation during the active vegetative growth stage. 

### Yield and its components

At physiological maturity, ten plants were randomly selected from each treatment and harvested to evaluate the effects of the applied inducers on crop productivity. Yield parameters assessed included pod length (cm), pod diameter (cm), number of pods per plant, and 100-seed weight (g). 

## Results

### Comparative inhibitory effects of bulk and nano-scale chemical inducers on *F. oxysporum* mycelial development

**Fig. 1:**
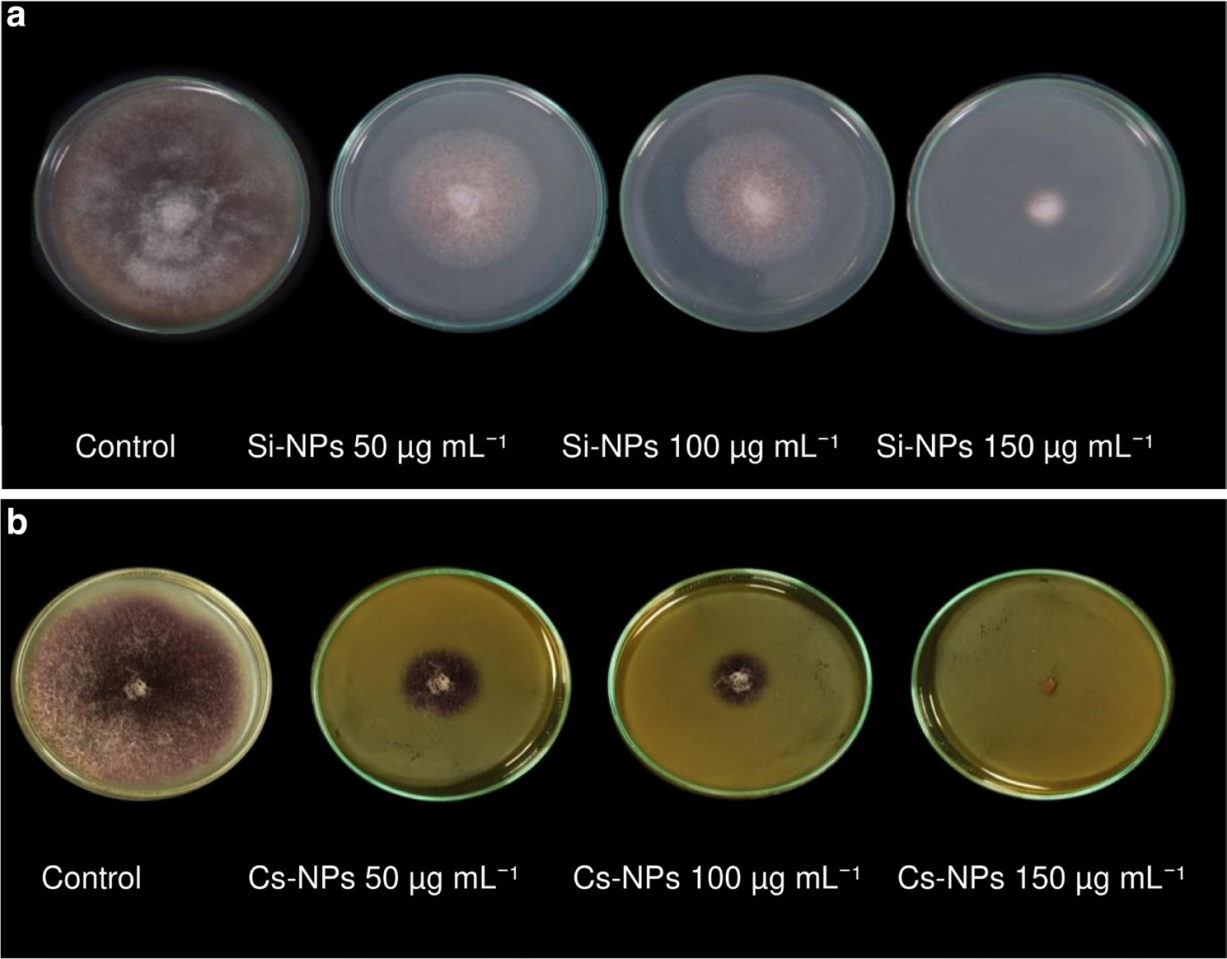
In vitro antifungal activity of silica and chitosan nanoparticles against *F. oxysporum*. Concentration-dependent effects of (**a**) Si-NPs and (**b**) Cs-NPs on radial mycelial growth and percentage of growth inhibition of *F. oxysporum* f. sp. *pisi*, assessed using the poisoned food technique. Both nano-formulations achieved complete inhibition (100%) of fungal growth at 150 µg mL⁻¹. The pronounced reduction in colony diameter highlights the strong antifungal efficacy of the nanoparticles, likely attributable to their high surface area and enhanced reactivity, facilitating direct interactions with fungal hyphae and disruption of cellular integrity

**Fig. 2:**
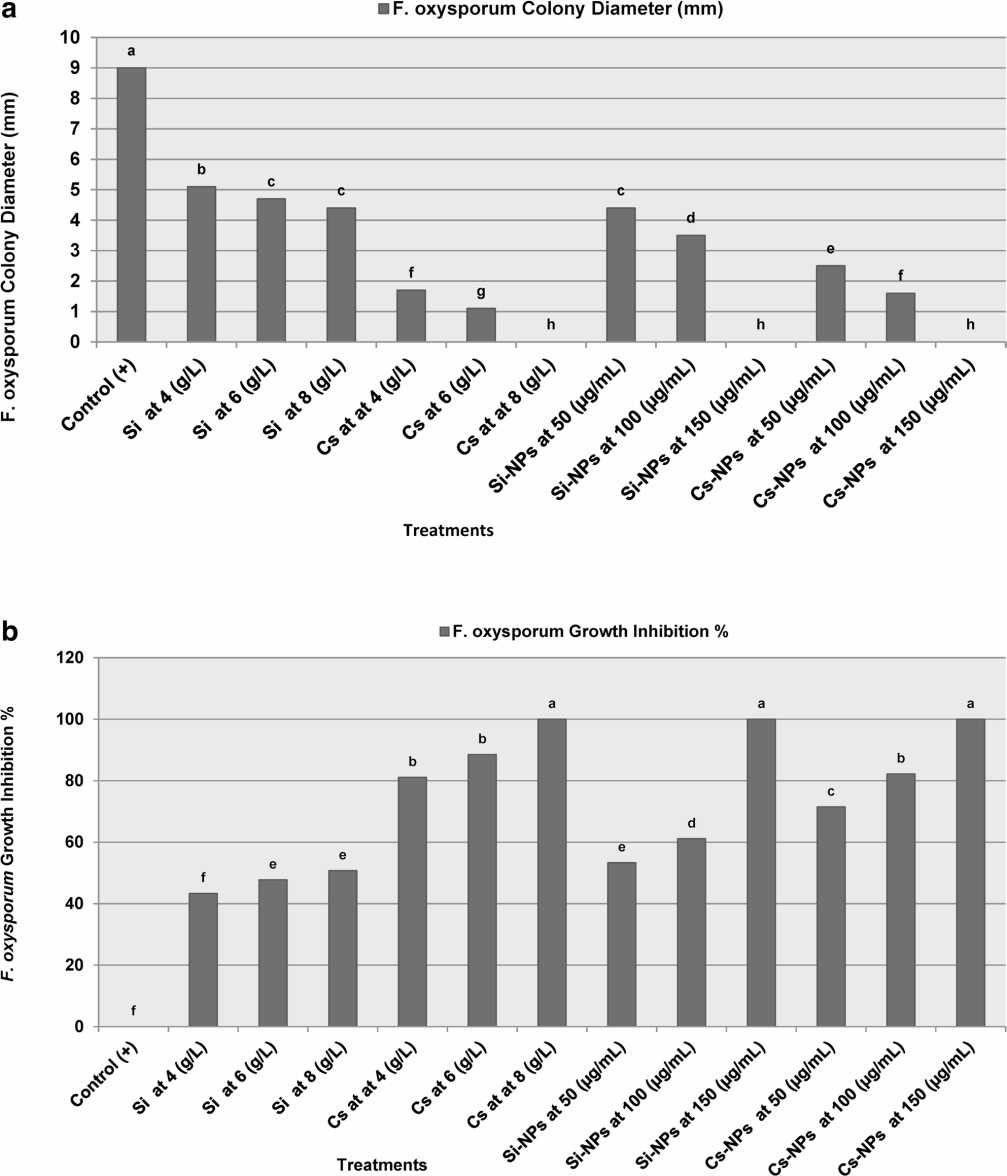
Comparative in vitro effects of conventional and nano-formulated silica and chitosan on *F. oxysporum* growth. (**a**) Radial colony diameter (mm) and (**b**) percentage of mycelial growth inhibition of *F. oxysporum* under different treatments. Data represent mean values, and columns sharing the same lowercase letter are not significantly different according to Duncan’s multiple range test (*P* ≤ 0.05). Si and Cs indicate silicon (applied as sodium metasilicate) and chitosan, respectively, while Si-NPs and Cs-NPs represent their corresponding nanoparticle formulations. The positive control refers to untreated plates inoculated with *F. oxysporum*

The antifungal activity of Si and Cs, in both conventional and nano-formulations, was evaluated against *F. oxysporum* using the poisoned food technique. The results (Figs. 1a and b and 2a and b) demonstrated that increasing the concentration of all treatments led to a progressive and significant reduction in radial mycelial growth. Among the tested formulations, Si-NPs and Cs-NPs exhibited the highest inhibitory efficacy, achieving complete suppression (100%) of mycelial growth at a concentration of 150 µg/mL. In contrast, conventional Si applied at 8 g/L achieved only 50.74% inhibition, whereas conventional Cs at the same concentration (8 g/L) resulted in complete growth inhibition. These findings highlight the superior antifungal potency of nano-formulations, which achieved comparable or higher efficacy at significantly lower concentrations than their conventional counterparts.

### Evaluation of chemical inducers against* F. oxysporum* under greenhouse conditions: 

#### Effect on Fusarium wilt severity

**Fig. 3:**
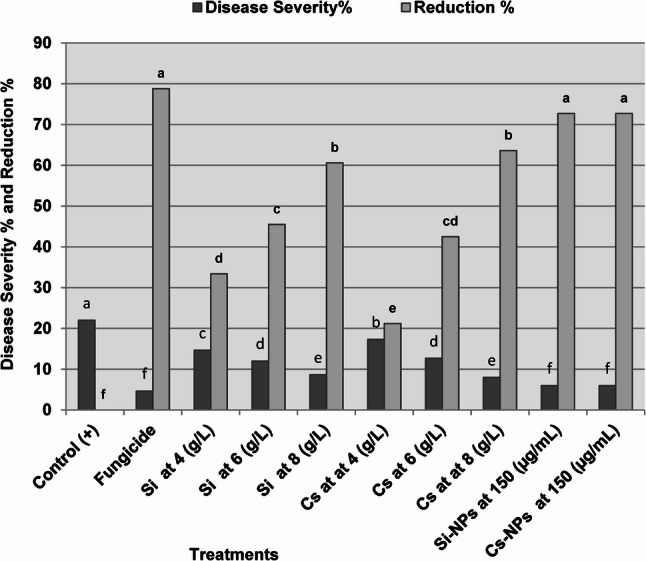
Suppression of Fusarium wilt severity in pea plants by conventional and nano-formulated treatments. Effects of silicon and chitosan applied at 4, 6, and 8 g L⁻¹, their nano-formulations (Si-NPs and Cs-NPs at 150 µg mL⁻¹), and the fungicide Topsin-M 70% WP on disease severity and disease reduction percentage under greenhouse conditions. Bars represent mean values, and different lowercase letters indicate significant differences according to Duncan’s multiple range test (*P* ≤ 0.05). The positive control represents *F. oxysporum*-infected untreated plants

As shown in Fig. 3, seed treatments with the tested chemical inducers significantly reduced Fusarium wilt severity compared with the untreated infected control. The greatest disease suppression was recorded for Si-NPs and Cs-NPs, both of which reduced wilt severity by 72.7%. This level of protection was comparable to that achieved by the fungicide Topsin-M 70% WP, which resulted in a 78.8% reduction in disease severity. Conventional silicon and chitosan applied at 8 g L⁻¹ also significantly decreased wilt severity by 60.6% and 63.6%, respectively. In contrast, lower concentrations (4–6 g L⁻¹) provided only partial disease reduction. Overall, nanoparticle-based treatments consistently exhibited higher efficacy than their corresponding conventional forms under greenhouse conditions.

#### Effect on total phenolic content and defense-related enzyme activities

**Fig. 4:**
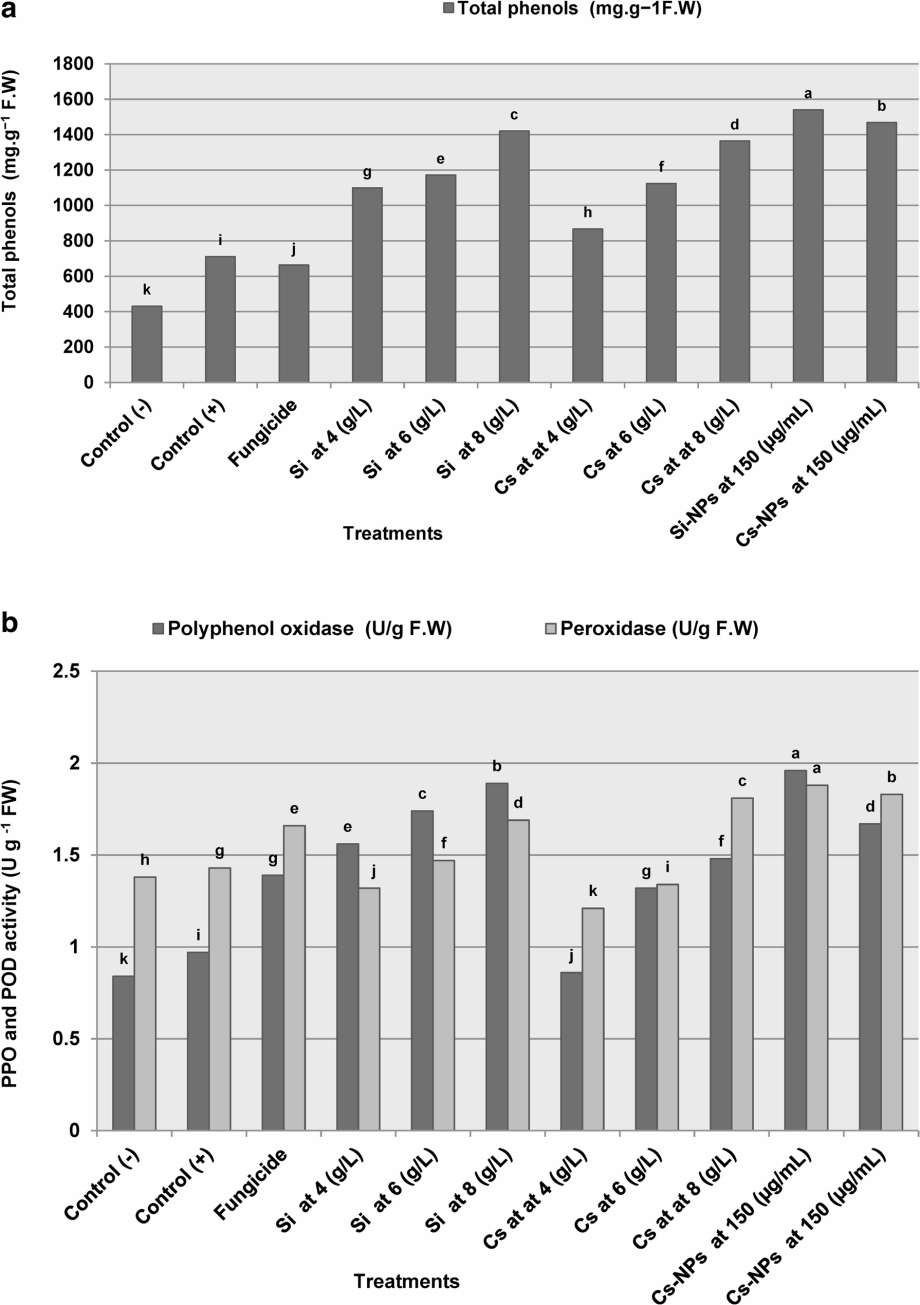
Induction of phenolic compounds and defense-related enzymes in pea plants following seed treatment with silica and chitosan formulations. (**a**) Total phenolic content and (**b**) activities of peroxidase (POD) and polyphenol oxidase (PPO) in pea plants infected with *F. oxysporum*. Bars represent mean values, and columns sharing the same lowercase letter are not significantly different according to Duncan’s multiple range test (*P* ≤ 0.05). Control (–) denotes healthy, uninfected plants, whereas Control (+) denotes infected untreated plants. Topsin-M 70% WP served as the fungicide reference treatment

Data presented in Fig. 4a, b clearly demonstrate that all tested chemical inducers significantly enhanced the defense-related enzyme activities in pea plants under greenhouse conditions compared to the untreated infested control. Among the treatments, Si-NPs exhibited the highest stimulatory effect, leading to remarkable increases in total phenolic content (116.8%), polyphenol oxidase (PPO; 102%), and peroxidase (POD; 31.4%). This was followed by Cs-NPs, which also showed substantial enhancements in total phenols (106.6%), PPO (72.1%), and POD (27.9%). Similarly, the conventional form of Si at 8 g/L significantly boosted these parameters, recording increases of 229.7% in total phenols, 125% in PPO, and 22.4% in POD, followed by conventional Cs at 8 g/L. These results suggest that nanoparticle formulations are more effective in priming the plant’s antioxidant defense system at much lower concentrations than their bulk counterparts.

#### Effect on photosynthetic pigments, membrane permeability, and lipid peroxidation

**Fig. 5:**
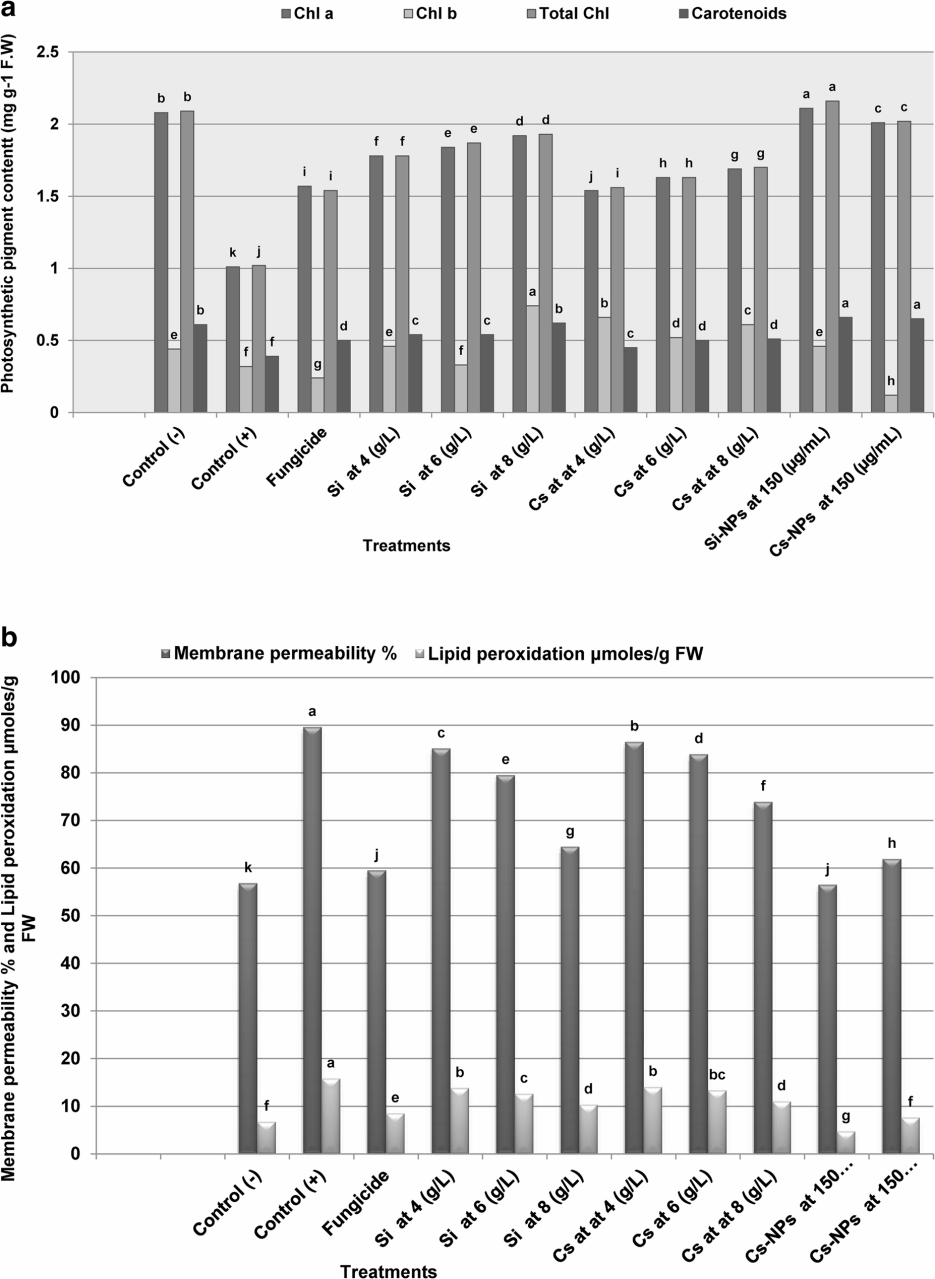
Effects of silica and chitosan seed treatments on photosynthetic pigments and membrane stability in pea plants under Fusarium wilt stress. (**a**) Photosynthetic pigment contents (chlorophyll a, chlorophyll b, total chlorophyll, and carotenoids) and (**b**) membrane permeability (%) and lipid peroxidation expressed as malondialdehyde (MDA; µmol g⁻¹ fresh weight). Bars represent mean values, and columns sharing the same lowercase letter are not significantly different according to Duncan’s multiple range test (*P* ≤ 0.05). Control (–) indicates healthy plants, while Control (+) represents infected untreated plants

Data presented in Fig. 5a, b revealed that seed treatments with chemical inducers markedly influenced photosynthetic pigments and stress-related physiological parameters in pea plants under *F. oxysporum* stress. Among all treatments, Si-NPs exerted the most significant positive impact, resulting in substantial increases of 108.9% in chlorophyll *a*, 43.75% in chlorophyll *b*, 111.7% in total chlorophyll, and 69.2% in carotenoids compared to the infested control. Furthermore, Si-NPs significantly mitigated cellular stress by reducing membrane permeability by 37.1% and lipid peroxidation (MDA content) by 69.8% (µmol g⁻¹ FW).

Similarly, Cs-NPs markedly enhanced photosynthetic efficiency, with increases of 99% in chlorophyll *a*, 15% in chlorophyll *b*, 98% in total chlorophyll, and 66.6% in carotenoids. These physiological improvements were accompanied by notable reductions in membrane permeability (31.1%) and lipid peroxidation (51.2% µmol g⁻¹ FW). While increasing concentrations of conventional silicon and chitosan also led to significant improvements in pigment content and membrane integrity, their efficacy remained consistently lower than that of the nano-formulations.

#### Effect on seed quality

**Fig. 6:**
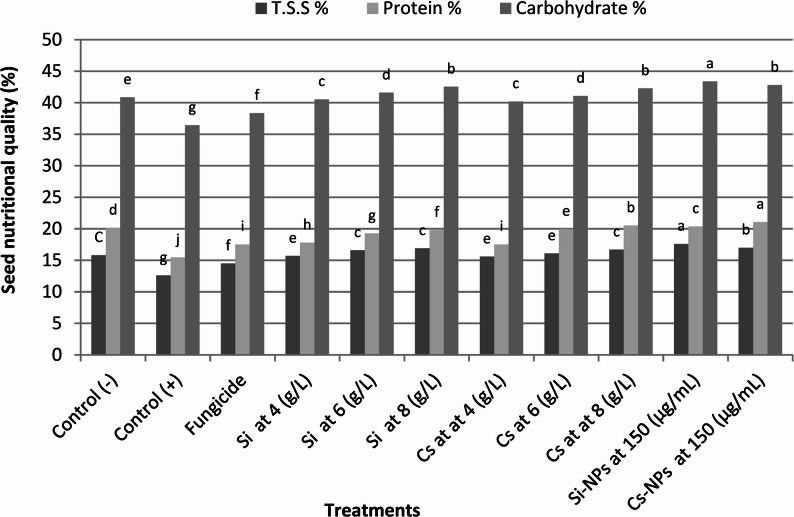
Effect of seed treatments with conventional and nano-formulated chemical inducers on seed quality parameters of pea plants infected with *F. oxysporum*. The figure illustrates the influence of different treatments on the major nutritional components of harvested seeds, including total protein and total carbohydrate contents. Treatments included silicon (Si) and chitosan (Cs) applied at 4, 6, and 8 g L⁻¹, their nano-formulations (Si-NPs and Cs-NPs at 150 µg mL⁻¹), and the fungicide control. Bars represent mean values, and columns sharing the same lowercase letter are not significantly different according to Duncan’s multiple range test (*P* ≤ 0.05). Si and Cs refer to silicon and chitosan, respectively, whereas Si-NPs and Cs-NPs denote their corresponding nanoparticle formulations. Control (–) represents healthy, uninfected, and untreated plants, while Control (+) represents *F. oxysporum*-infected but untreated plants. The fungicide treatment consisted of Topsin-M 70% WP), which served as the standard positive control

As shown in Fig. 6, all nanoparticle (NP) seed treatments significantly improved the quality parameters of pea seeds under *F. oxysporum* infection compared with the untreated infected control. Among the treatments, Si-NPs had the strongest impact, resulting in substantial increases in total soluble solids (39.6%), protein content (32.3%), and carbohydrate content (18.9%). This was followed by Cs-NPs, which enhanced total soluble solids, protein, and carbohydrate contents by 34.9%, 36.2%, and 17.3%, respectively. Overall, the nanoparticle formulations were more effective than their conventional counterparts in enhancing seed quality. At the highest applied concentration, silicon (8 g/L) also improved seed quality, increasing total soluble solids, protein, and carbohydrate contents by 34.1%, 27.8%, and 16.6%, respectively. Cs(8 g/L) showed similar but slightly lower improvements compared with its nano-form.

#### Effect on pea roots structure

**Fig. 7:**
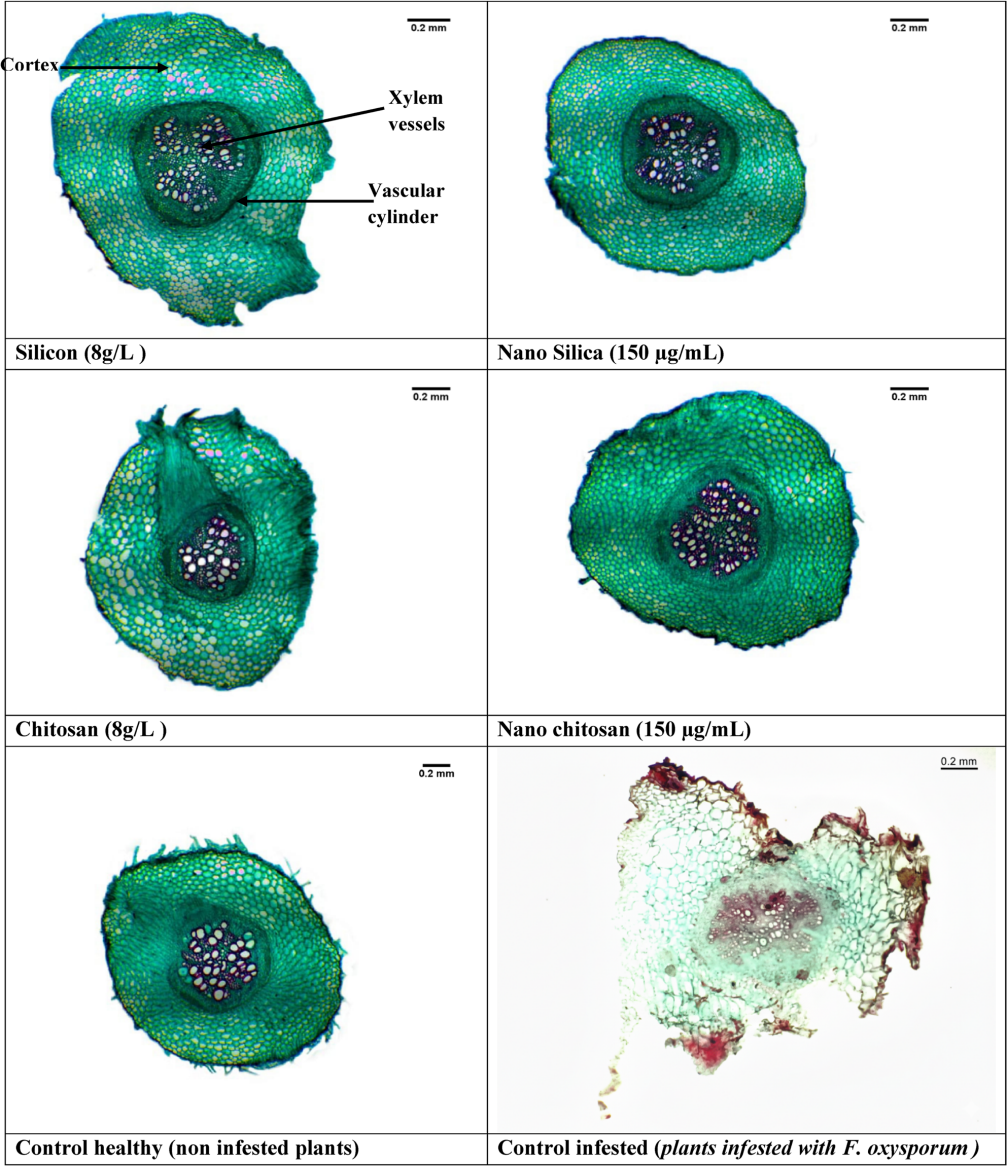
Anatomical alterations in pea root tissues induced by silica and chitosan formulations under *F. oxysporum* infection. Transverse root sections (×100) illustrating histological responses to Si, Si-NPs, Cs, and Cs-NPs compared with the infected control. Treatments, particularly nano-formulations, markedly enhanced cortical thickness, vascular cylinder development, and xylem vessel diameter. These structural modifications suggest reinforced mechanical barriers and improved vascular functionality, contributing to enhanced tolerance against Fusarium wilt

Results presented in Table 1 and Fig. 7 show that *F. oxysporum* infection caused a marked reduction in pea root anatomical characteristics compared with the healthy control. Seed treatments with silicon- and chitosan-based inducers significantly alleviated these negative effects and improved root structural traits. Application of conventional silicon (8 g L⁻¹) increased total root section thickness by 6.42%, mainly due to increases in cortex thickness (8.24%) and vascular cylinder thickness (8.65%), while xylem vessel diameter increased by 82.55% relative to the infected control. Similarly, chitosan (8 g L⁻¹) enhanced total section thickness by 11.64% and vascular cylinder thickness by 20.01%, accompanied by a 50.34% increase in xylem vessel diameter. Nano-formulations exhibited the most pronounced effects on root anatomy. Si-NPs (150 µg mL⁻¹) produced the highest increases in total root section thickness (15.81%), cortex thickness (12.51%), and vascular cylinder thickness (21.00%), along with a marked enlargement of xylem vessel diameter (90.60%) compared with the infected control. Likewise, Cs-NPs (150 µg mL⁻¹) significantly improved section, cortex, and vascular cylinder thickness by 13.73%, 7.85%, and 19.02%, respectively, and increased xylem vessel diameter by 63.76%. In addition, nano-treatments modified the cortex-to-vascular cylinder thickness ratio, reflecting a more balanced distribution of protective and conductive tissues compared with the infected untreated plants.

**Table 1:** Effect of chemical inducers seed treatments on anatomical features of pea infested roots with *F. oxysporum*

Treatment	Characters of root anatomy								
	Section thickness		Cortex thickness		Vascular cylinder thickness		Xylem vessels diameter		(Cortex Thickness /Vascular cylinder thickness) *100
	µ	± % to control	µ	± % to control	140.74	± % to control	µ	± % to control	
Control (-)	1425.43	6.84	744.26	0.26	701.33	20.27	47.5	59.40	106.12
Control (+)	1334.15	0	742.35	0	583.15	0	29.8	0	127.3
Silicon at 8 g/L	1419.84	6.42	803.52	8.24	633.6	8.65	54.4	82.55	126.8
Chitosan at 8 g/L	1489.44	11.64	789.12	6.30	699.84	20.01	44.8	50.34	112.75
Si-NPs 150 µg/mL	1545.12	15.81	835.2	12.51	705.6	21.00	56.8	90.60	118.36
Cs-NPs 150 µg/mL	1517.28	13.73	800.64	7.85	694.08	19.02	48.8	63.76	115.33

Si and Chit refer to silicon (as sodium meta-silicate) and chitosan, respectively, whereas Si-NPs and Cs-NPs denote their corresponding nanoparticle formulations. Control (−) indicates healthy, uninfected, and untreated plants, while Control (+) represents *F. oxysporum*-infected but untreated plants. Fungicide refers to Topsin-M 70% WP, which served as the standard positive control for disease management

#### Effect on growth parameters

Data presented in Table 2 clearly indicate that seed priming with nano-chemical inducers markedly enhanced vegetative growth performance compared with the infected control. The untreated infected plants exhibited pronounced reductions in shoot and root length, leaf area, and both fresh and dry weights due to the vascular blockage and metabolic disruption caused by *F. oxysporum* infection.

**Table 2:** Effect of seed treatments with chemical inducers on vegetative growth parameters of pea plants infected with *F. oxysporum*

Treatment	Shoot length (cm)	Root length (cm)	Leaf area (cm)^2^	Shoot fresh weight (g)	Shoot dry weight (g)	Root fresh weight (g)	Root Dry weight (g)
Control (+)	27^g^	5.7^f^	45.27^j^	2.43^j^	0.93^j^	0.15^f^	0.02^f^
Control (-)	37.8^bc^	7.6 ^bcde^	115.17^b^	5.71^d^	1.46^d^	0.31^b^	0.08^ab^
Fungicide	34.7^d^	7.3^cde^	96.09^f^	5.29^f^	1.31^g^	0.35^a^	0.06^cde^
Si at 4 g/L	35.7^d^	6.7^e^	101.19^e^	5.17^g^	1.51^f^	0.23^d^	0.05^de^
Si at 6 g/L	38.7^bc^	7.0^de^	110.00^d^	5.34^e^	1.53^e^	0.24^a^	0.06^cde^
Si at 8 g/L	39^bc^	8.7^ab^	116.30^b^	6.06^c^	1.81^b^	0.29^c^	0.07^abc^
Chit at 4 g/L	30^f^	6.7^e^	51.35^i^	2.79^i^	1.02^i^	0.18^e^	0.04^e^
Chit at 6 g/L	31.7^e^	7.7^bcde^	58.27^h^	4.17^h^	1.19^h^	0.22^d^	0.07^bcd^
Chit at 8 g/L	38.3^c^	8.0^bcd^	75.86^g^	5.72^d^	1.57^d^	0.28^c^	0.09^ab^
Si-NPs at 150 µg/mL	41.3^a^	9.3^a^	124.69^a^	6.62^a^	2.05^a^	0.34^a^	0.09^ab^
Cs-NPs 150 µg/mL	40^b^	8.3^bc^	115.37^c^	6.47^b^	1.78^c^	0.31^b^	0.09^ab^

Values within each column followed by the same letter are not significantly different according to Duncan’s multiple range test (*P* ≤ 0.05). Si and Chit refer to silicon (as sodium metasilicate) and chitosan, respectively, whereas Si-NPs and Cs-NPs denote their corresponding nanoparticle formulations. Control (−) indicates healthy, uninfected, and untreated plants, while Control (+) represents *Fusarium oxysporum*–infected but untreated plants. Fungicide refers to Topsin-M 70% WP, which served as the standard positive control for disease management

Among the treatments, Si-NPs achieved the greatest enhancement across all vegetative traits, recording a 53.2% increase in shoot length and a 49.7% increase in root length compared to the infected control. This was closely followed by Cs-NPs, which improved these parameters by approximately 48.6% and 45.2%, respectively. Conventional formulations of Si and Cs also promoted plant growth but to a lesser extent, indicating that nanoscale delivery significantly improved uptake, translocation, and bioactivity within plant tissues.

#### Effect on yield parameters

**Fig. 8:**
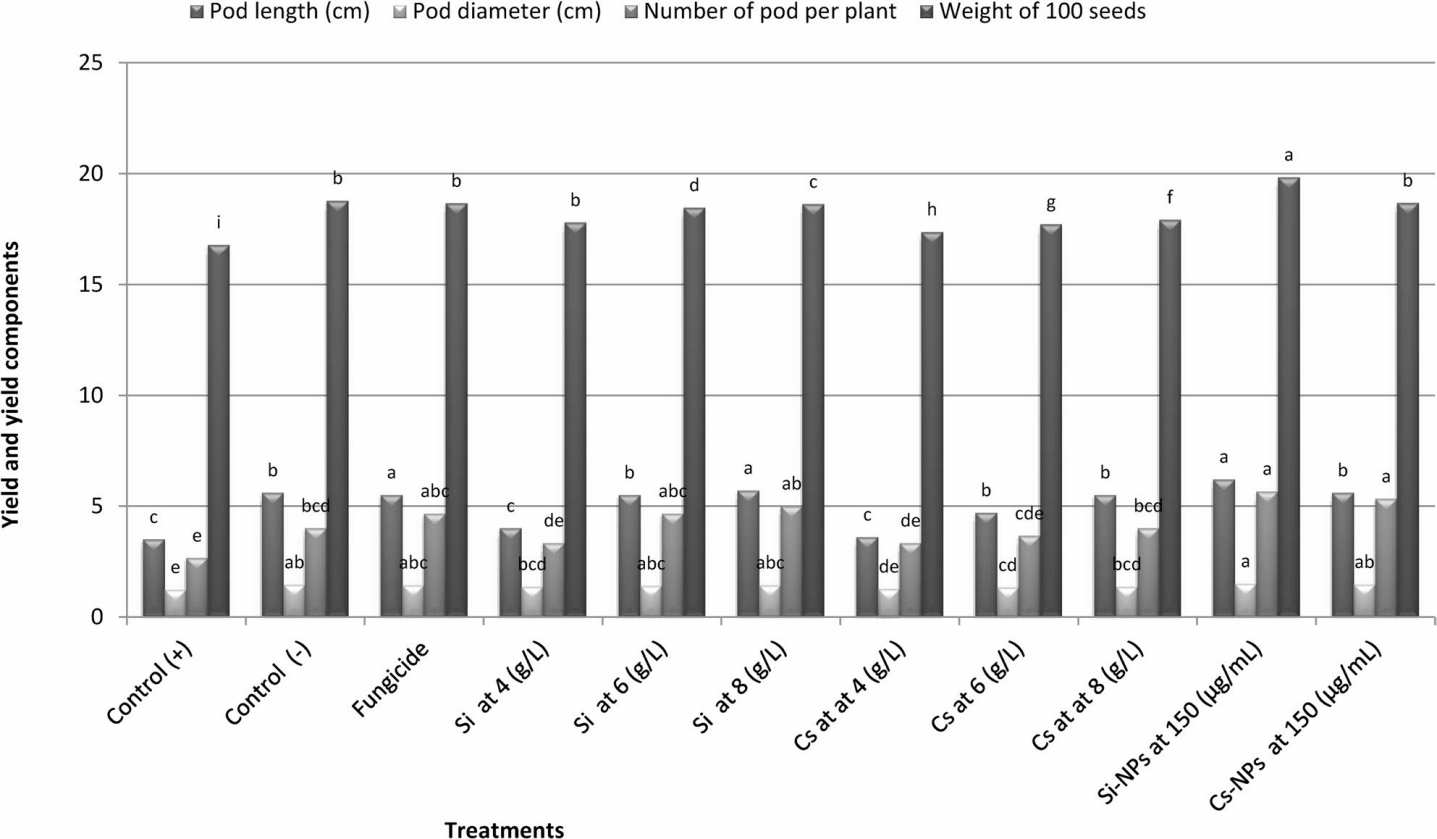
Improvement of yield-related traits in pea plants by silica and chitosan formulations under Fusarium wilt conditions. Effects of seed treatments on pod length (cm), pod diameter (cm), number of pods per plant, and 100-seed weight (g). Bars represent mean values, and columns sharing the same lowercase letter are not significantly different according to Duncan’s multiple range test (*P* ≤ 0.05). Control (–) indicates healthy untreated plants, whereas Control (+) denotes infected untreated plants. Topsin-M 70% WP was used as the standard fungicide reference

As shown in Fig. 8, the application of nano-inducers substantially improved yield attributes of pea plants compared with both infected and untreated controls. *F. oxysporum* infection drastically reduced pod length, pod diameter, pod number per plant, and seed weight due to vascular obstruction, impaired photosynthate allocation, and premature senescence.

Treatment with Si-NPs resulted in the most significant recovery of yield components, with a 54.5% increase in pod length, 50.7% increase in pod diameter, and 58.4% increase in 100-seed weight relative to the infected control. Cs-NPs ranked second, improving the same parameters by 48–52%, demonstrating their efficiency in sustaining reproductive development under pathogen pressure. Meanwhile, bulk Si and Cs treatments provided moderate protection, suggesting that nanoscale formulations achieved superior systemic distribution and induced stronger biochemical defenses.

## Discussion

The present study demonstrates that silicon and chitosan, particularly in their nano-formulations, exert a strong suppressive effect on *F. oxysporum* in pea plants. In vitro assays revealed that Si-NPs and Cs-NPs completely inhibited mycelial growth at 150 µg/mL, whereas their conventional forms were markedly less effective. This superior antifungal activity can be attributed to the unique physicochemical properties of nanoparticles, including their high surface-to-volume ratio, enhanced reactivity, and improved dispersibility, which facilitate close interaction with fungal cell walls and membranes, ultimately leading to structural disruption and metabolic impairment [38, 39]. In addition, nanoparticles may induce localized oxidative stress through ROS generation, causing damage to proteins, nucleic acids, and lipids, thereby impairing fungal viability [40]. This combined physical and biochemical mode of action likely explains the complete growth inhibition observed. Similar antifungal effects of Si-NPs have been reported in tomato and cucumber systems under disease pressure [23, 41], while Cs-NPs are known to penetrate fungal cell walls more efficiently than bulk chitosan, binding to negatively charged surfaces and increasing membrane permeability [21].

Consistent with the in vitro findings, seed treatment with chemical inducers—particularly in nano-form—significantly reduced Fusarium wilt severity under greenhouse conditions. Both Si-NPs and Cs-NPs lowered disease severity by approximately 72.7%, achieving levels of protection comparable to the systemic fungicide Topsin-M 70% WP. This outcome highlights the practical relevance of nano-inducers as eco-friendly alternatives to synthetic fungicides. Silicon-mediated disease suppression operates through both mechanical reinforcement of plant tissues and modulation of defense-related gene expression. These findings are consistent with previous reports demonstrating reduced *F. oxysporum* and other fungal infections following silicon nanoparticle application [42–44]. Similarly, chitosan nanoparticles have demonstrated effective control of *F. oxysporum*–induced wilt in other legumes, indicating their broad-spectrum antifungal potential [22].

The resistance induced by silicon is largely associated with reinforcement of cell wall architecture, which limits pathogen penetration, alongside activation of biochemical defense responses, including the induction of antioxidant enzymes and antimicrobial metabolites [45]. Although silicon may exhibit some direct antifungal effects [46], its primary contribution lies in promoting callose and lignin deposition and enhancing antioxidant capacity, thereby alleviating pathogen-induced oxidative stress [47, 48]. Nano-formulated silicon further amplifies these responses by improving uptake, mobility, and bioavailability within plant tissues.

Chitosan-mediated antifungal activity is mainly driven by its electrostatic interaction with negatively charged fungal cell surfaces, resulting in increased membrane permeability and growth inhibition [49]. Chitosan also promotes ROS accumulation, leading to oxidative damage and inhibition of spore germination [50]. Microscopic observations from previous studies have confirmed severe hyphal deformation following chitosan exposure [50–52]. In parallel, chitosan acts as a potent elicitor of systemic resistance by activating salicylic acid (SA)- and jasmonic acid (JA)-dependent signaling pathways, which enhance the accumulation of pathogenesis-related proteins and phenolic compounds [21, 53]. The nano-formulation of chitosan enhances these effects through increased solubility and more efficient interaction with plant cell surfaces.

Biochemical analyses in the present study provide strong evidence that nano-inducers prime pea plant defenses against *F. oxysporum*. Treatment with Si-NPs markedly increased total phenolic content and significantly enhanced the activities of POD and PPO, enzymes involved in lignin biosynthesis and phenolic polymerization that contribute to cell wall fortification and pathogen restriction [54]. Concurrent reductions in lipid peroxidation and membrane permeability further indicate effective mitigation of oxidative damage and stabilization of cellular membranes. This biochemical profile is characteristic of a primed defense state, in which elicitor-treated plants exhibit heightened readiness to respond rapidly and effectively upon pathogen challenge [55, 56].

The observed increases in chlorophyll a, chlorophyll b, total chlorophyll, and carotenoid contents following Si-NP and Cs-NP treatments further support their protective role. In addition to sustaining photosynthetic performance, these pigments contribute to redox homeostasis under stress conditions, with carotenoids functioning as key antioxidants that protect chloroplast membranes from ROS-induced damage [57]. Similar protective effects of nanoparticle treatments on photosynthesis and antioxidant capacity have been reported in various crops under biotic stress [23, 39].

Anatomical investigations in the present study provided strong evidence that Si-NPs and Cs-NPs play a pivotal role in reinforcing pea root structures against F. oxysporum. Application of these nano-inducers significantly increased cortex and vascular cylinder thickness, accompanied by a marked enlargement of xylem vessel diameter. These changes represent an integrated defense strategy rather than simple structural alterations. Cortex thickening likely functions as a physical barrier limiting pathogen penetration, while enhanced vascular development supports sustained water and nutrient transport, processes commonly disrupted during vascular wilt infection. The increased xylem vessel diameter may further improve hydraulic conductivity, compensating for vessel blockage caused by fungal colonization. Such anatomical fortification is widely recognized as a hallmark of induced resistance in plants [58].

Importantly, the cortex-to-vascular cylinder thickness ratio provided additional insight into tissue-level defense allocation. Although both nano-formulations increased absolute tissue thickness, Si-NPs optimized this ratio, suggesting a balanced reinforcement of protective outer tissues without excessive restriction of the vascular core. This coordinated anatomical adjustment may enhance resistance while maintaining physiological efficiency, a critical requirement for long-term disease tolerance. In contrast, conventional treatments showed less pronounced or less harmonized anatomical responses, highlighting the superior efficacy of nano-formulations in modulating root architecture.

Silicon deposition within the apoplast and cell walls promotes lignification and callose accumulation, strengthening mechanical barriers against pathogen ingress and regulating genes associated with cell wall biosynthesis and vascular differentiation [14, 48, 56, 59, 60]. Likewise, chitosan stimulates phenolic deposition and lignin-related enzymatic activity, further restricting pathogen spread within vascular tissues [21, 53, 60]. These anatomical responses are consistent with previous reports demonstrating that nanoparticle treatments enhance vascular resilience and nutrient transport under both biotic and abiotic stresses [23, 47].

Collectively, the biochemical and anatomical responses observed in this study indicate that Si-NPs and Cs-NPs confer resistance through a dual mechanism involving structural reinforcement and physiological optimization. These integrated responses not only restrict pathogen development but also sustain photosynthetic efficiency, biomass accumulation, and reproductive performance.

The growth-promoting effects observed under pathogen pressure likely reflect the combined influence of reduced disease burden and direct enhancement of plant physiological processes. Silicon has been shown to improve root architecture, water uptake, and nutrient assimilation through regulation of aquaporins and transporter proteins [14], whereas chitosan can modulate phytohormone signaling pathways associated with cell division and elongation [53].

Yield attributes, including pod characteristics and seed weight, were substantially improved by nano-inducer treatments, particularly Si-NPs. These improvements can be attributed to enhanced photosynthetic efficiency, delayed senescence, and improved assimilate partitioning under stress conditions [14, 23, 47, 60]. In parallel, the observed enhancement in seed quality parameters, including protein, carbohydrate content, and total soluble solids, suggests that nano-inducers positively influence carbon and nitrogen metabolism during seed development. Similar improvements in seed nutritional quality following silicon and chitosan application have been reported in other crops [16, 60].

The results of this study demonstrated that Si-NPs and Cs-NPs markedly enhanced vegetative growth of pea plants under both greenhouse and field conditions, as evidenced by significant increases in shoot and root length, leaf area, branching, and biomass accumulation. Among all treatments, Si-NPs consistently exhibited the strongest growth-promoting effect. These improvements can be attributed to the dual functionality of nanoparticles, which simultaneously reduce pathogen pressure and directly stimulate plant physiological processes. Silicon is known to enhance root architecture, water uptake, and nutrient assimilation through the regulation of aquaporins and transporter proteins [14], while chitosan can modulate phytohormone signaling—particularly auxin and cytokinin pathways—thereby promoting cell division and elongation [53]. The nano-formulation likely enhances the bioavailability and transport efficiency of these inducers, amplifying their positive impact on plant growth.

Yield components, including pod length, pod diameter, number of pods per plant, pod weight, and hundred-seed weight, were significantly enhanced by nano-inducer treatments, particularly Si-NPs. Yield improvements exceeded 70% for certain parameters compared with the control and were comparable to, or even surpassed, those achieved by fungicide treatments. These yield enhancements can be attributed not only to reduced disease severity but also to improved photosynthetic efficiency and assimilate partitioning. Silicon is known to delay leaf senescence, enhance chlorophyll retention, and improve carbon assimilation under stress conditions [14, 23, 47, 60]. Similarly, chitosan has been reported to stimulate carbohydrate metabolism and assimilate translocation, resulting in improved pod development and seed filling [21, 60]. These findings are consistent with previous studies demonstrating that Si-NPs and Cs-NPs significantly enhance crop productivity under pathogen stress [14, 39].

In addition to yield improvement, nano-formulations markedly enhanced seed quality parameters, including total soluble solids (TSS), protein content, and carbohydrate content of pea seeds. Si-NPs increased protein content by 32% and carbohydrate content by nearly 19%, while Cs-NPs produced comparable improvements. These results indicate that nano-inducers not only suppress disease but also promote metabolic processes associated with nutrient accumulation in seeds. The enhanced protein and carbohydrate contents may be attributed to improved nitrogen and carbon metabolism mediated by silicon and chitosan, as well as their roles in maintaining membrane integrity and minimizing oxidative losses during seed development [14, 60]. Similar positive effects of Si-NPs on seed quality have been reported in various crops, where improved mineral nutrition and stress mitigation contributed to higher grain protein and carbohydrate contents [16].

The safety and biocompatibility of nanomaterials are critical considerations for their adoption in sustainable agriculture [61]. In the present study, pea plants treated with Si-NPs and Cs-NPs exclusively via seed priming exhibited no visible phytotoxic effects throughout the experimental period. Typical symptoms of phytotoxicity, such as chlorosis, necrosis, leaf deformation, or growth inhibition, were not observed. Instead, treated plants showed enhanced seed germination, increased shoot and root biomass, and improved overall vigor compared with untreated controls, indicating that the applied nano-formulations acted as growth-promoting and resistance-inducing agents rather than physiological stressors [62, 63]. These positive responses are likely associated with the nano-specific characteristics of the applied materials, including controlled particle size, high surface area, and effectiveness at low application rates, which enhance bioavailability while minimizing cellular stress.

Beyond plant safety, potential human health risks represent an important aspect of nanomaterial application. Chitosan is a biodegradable and biocompatible polymer classified as Generally Recognized as Safe (GRAS), supporting its suitability for agricultural and food-related applications [64]. Likewise, silica-based nanomaterials have been widely reported as biologically safe at environmentally relevant concentrations. Notably, previous cytotoxicity studies conducted by our research group demonstrated that silica nanoparticles with comparable physicochemical properties were non-toxic to human lung (BEAS-2B) and kidney (Vero) epithelial cell lines at concentrations exceeding those used in the present study, exhibiting high IC₅₀ values and a broad safety margin [14]. Nevertheless, further long-term and field-scale investigations are required to assess nanoparticle persistence, translocation, and potential accumulation in edible plant tissues and soil ecosystems, thereby ensuring comprehensive environmental and consumer safety [65].

Future studies should focus on translating the promising greenhouse performance of Si-NPs and Cs-NPs into field-scale applications, with particular emphasis on their use as seed treatments under practical agricultural conditions. Multi-location field trials will be essential to validate their consistency, robustness, and cost-effectiveness across different agro-climatic zones. In addition, further research is required to optimize formulation stability, storage conditions, and large-scale application strategies to support potential commercialization. Assessing the long-term fate of these nano-formulations in soil systems and their interactions with native soil microbiota will also be critical to ensure environmental safety and sustainable integration into integrated disease management programs.

**Fig. 9:**
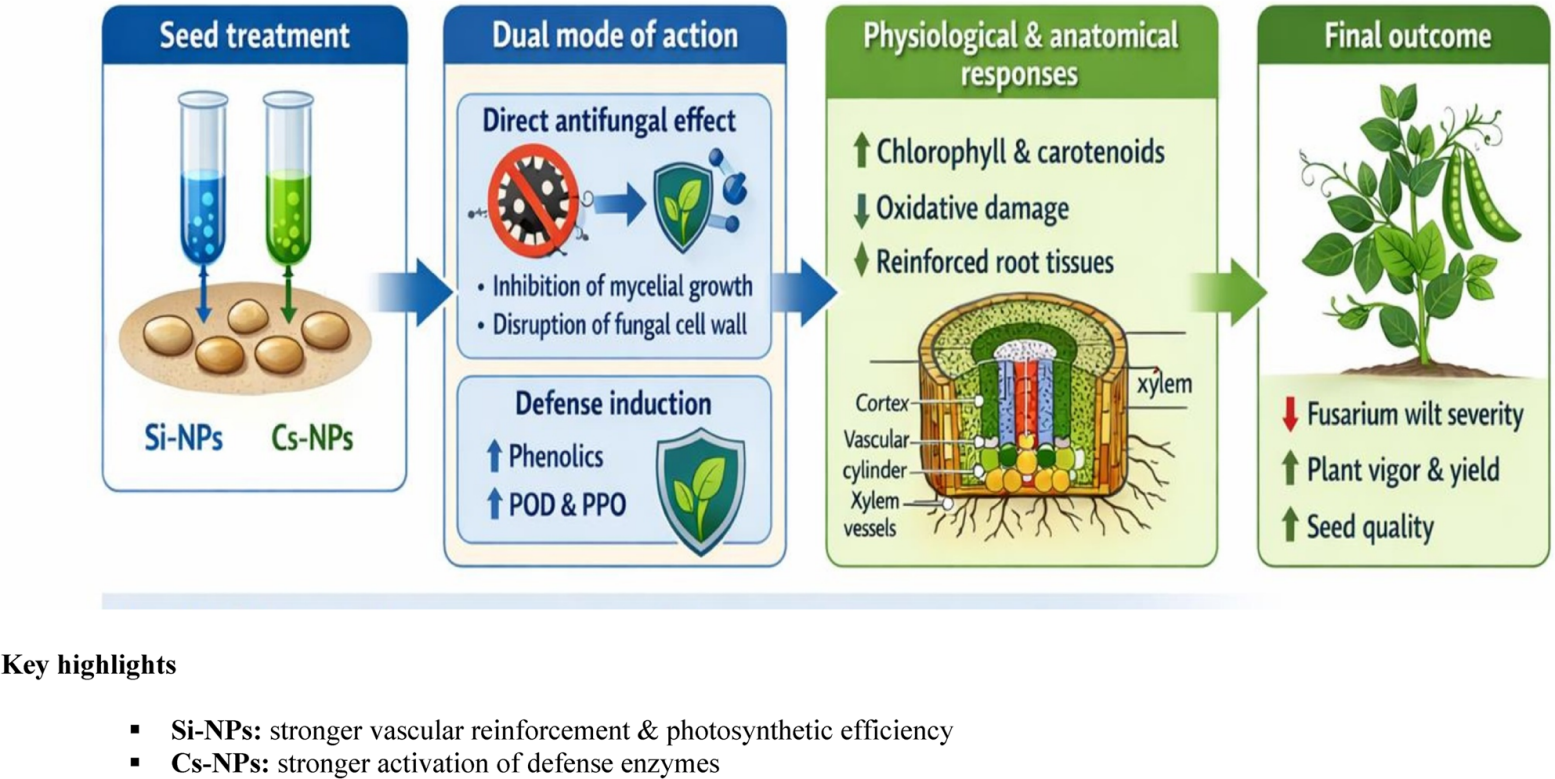
Proposed mechanistic model illustrating how seed treatment with silica and chitosan nanoparticles enhances resistance of pea plants against F. oxysporum. The model integrates direct antifungal effects with the induction of host defense responses, improved physiological performance, and anatomical reinforcement of root tissues. Si-NPs predominantly enhanced vascular development, photosynthetic efficiency, and oxidative stress mitigation, whereas Cs-NPs mainly stimulated defense-related enzymes and phenolic metabolism. These coordinated responses ultimately resulted in reduced disease severity and improved plant productivity

Based on the integrated biochemical, physiological, and anatomical evidence, a mechanistic model is proposed to explain the enhanced resistance of pea plants to *F. oxysporum* following seed treatment with silica nanoparticles (Si-NPs) and chitosan nanoparticles (Cs-NPs) (Fig. 9). The model highlights a dual mode of action combining direct antifungal activity with activation of host defense responses. Si-NPs and Cs-NPs directly suppress fungal growth, likely through disruption of hyphal cell walls and membranes, thereby limiting early pathogen establishment. Simultaneously, nanoparticle treatments induce systemic resistance in pea plants, as reflected by increased accumulation of phenolic compounds, enhanced activities of defense-related enzymes (PPO and POD), and reduced membrane damage and lipid peroxidation.

At the anatomical level, nano-formulations promote reinforcement of root tissues, including increased cortex thickness, vascular cylinder development, and xylem vessel enlargement. These structural modifications strengthen physical barriers against pathogen invasion while maintaining efficient water and nutrient transport, supporting improved physiological performance and photosynthetic capacity.

As summarized in Fig. 9, the coordinated antifungal, biochemical, and anatomical effects of Si-NPs and Cs-NPs collectively result in reduced Fusarium wilt severity, enhanced plant vigor, and improved yield and seed quality, providing a mechanistic basis for their superior efficacy compared with conventional formulations.

## Conclusion

This study demonstrates that silicon and chitosan, particularly in their nanoparticle forms (Si-NPs and Cs-NPs), are highly effective in controlling Fusarium wilt of pea caused by *F. oxysporum* f. sp. *pisi*. The nano-formulations exhibited strong antifungal activity in vitro and significantly reduced disease severity under greenhouse and field conditions, while simultaneously enhancing plant growth, yield, and seed quality.

The superior performance of Si-NPs and Cs-NPs compared with their bulk counterparts is attributed to their enhanced bioavailability and reactivity, enabling a dual mode of action involving direct pathogen suppression and activation of host defense mechanisms. Their efficacy was comparable to that of the synthetic fungicide Topsin-M 70% WP, without associated environmental or toxicological risks, highlighting their potential as sustainable, eco-friendly alternatives for integrated Fusarium wilt management in pea cultivation.

## Supplementary Information

Supplementary Material 1.

## Data Availability

The data supporting the findings of this study are available upon reasonable request from the corresponding author.

## Abbreviations

Si: Silicon (bulk form)
Si-NPs: Silicon nanoparticles
Cs: Chitosan (bulk form)
Cs-NPs: Chitosan nanoparticles
NP: Nanoparticle
PPO: Polyphenol oxidase
POD: Peroxidase
TSS: Total soluble sugars
MDA: Malondialdehyde
ELP: Electrolyte leakage percentage
PDA: Potato dextrose agar
TBA: Thiobarbituric acid
DAS: Days after sowing
DS: Disease severity
ARC: Agricultural Research Center
WP: Wettable powder
ANOVA: Analysis of variance
PR: Pathogenesis-related proteins
SAR: Systemic acquired resistance
ISR: Induced systemic resistance
ROS: Reactive oxygen species
FAA: Formalin–acetic acid–alcohol
GAE: Gallic acid equivalent
SA: Salicylic acid
JA: Jasmonic acid
SEM: Scanning electron microscope
MIC: The minimum inhibitory concentration
MP %: Membrane permeability
ELP: Electrolyte leakage percentage
TSS: Total soluble solids
