# Supplementary material for: Comparative efficacy of conventional and nano-formulations of silica and chitosan against Fusarium oxysporum causing wilt in pea (Pisum sativum)
Source: BMC Plant Biol. 2026 Mar 7;26:634. doi: 10.1186/s12870-026-08334-y (PMC13064303; doi:10.1186/s12870-026-08334-y)
Supplement: Supplementary file 1 — Supplementary Material 1. [file 12870_2026_8334_MOESM1_ESM.docx]

**Fig. S1. Characterization of the synthesized Si-NPs.**

(A) Transmission Electron Microscopy (TEM) micrograph of silicon nanoparticles (Si-NPs) showing a uniform spherical morphology and an average particle size of 20 nm. (B) X-ray diffraction (XRD) pattern of Si-NPs exhibiting a broad characteristic peak, which confirms its amorphous nature**.**


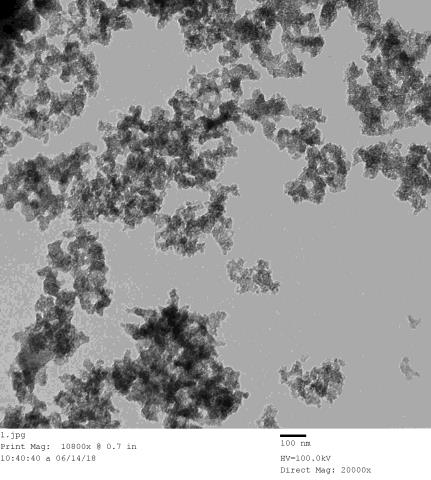

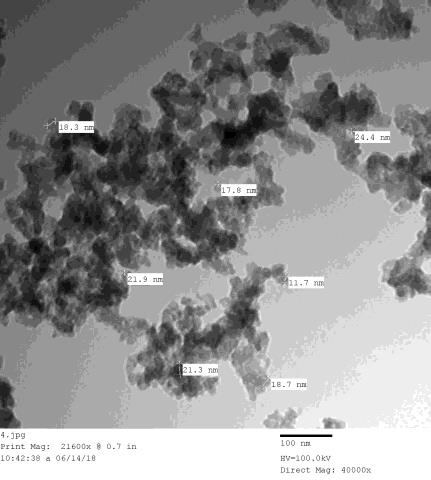


Fig. S1A.

**SiO2**

Fig. S1B

**Fig. S2. Physical properties and structural analysis of chitosan nanoparticles (Cs-NPs). (a) Dynamic light scattering (DLS) analysis showing the hydrodynamic size distribution of Cs-NPs with a mean diameter of 15 ± 3 nm and high monodispersity. (b) X-ray diffraction (XRD) pattern of Cs-NPs showing the specific crystalline peaks that confirm the successful synthesis and purity of the nano-chitosan formulation**.


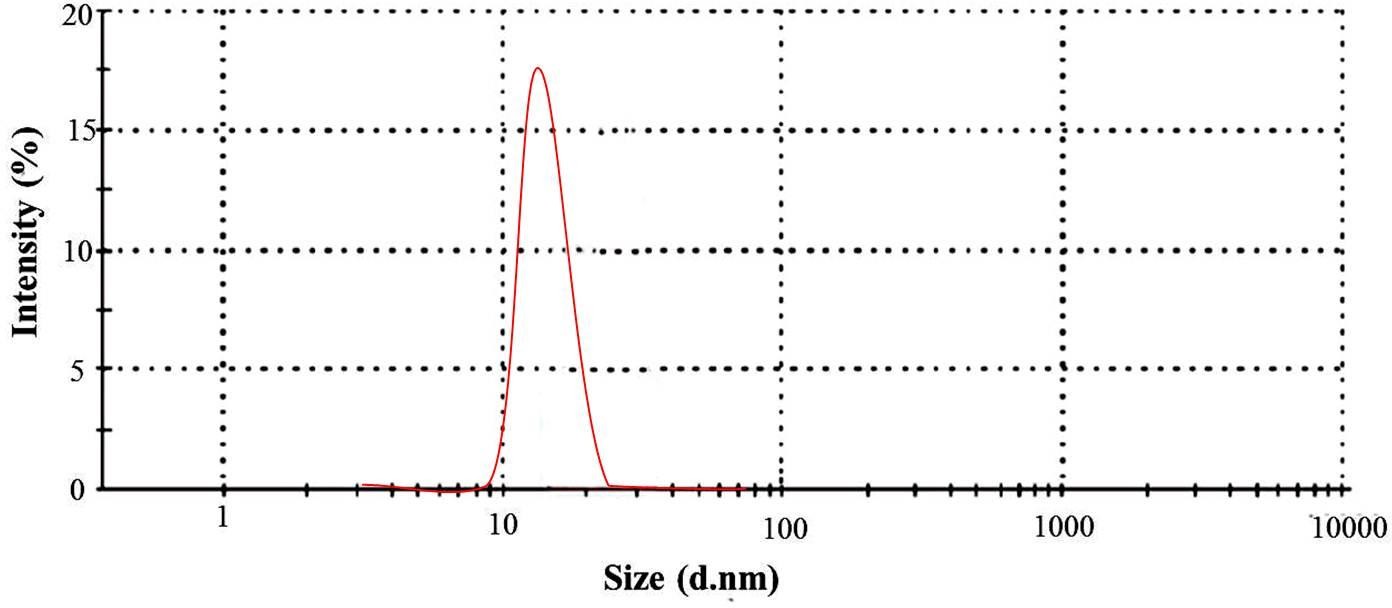


Fig. S2A.


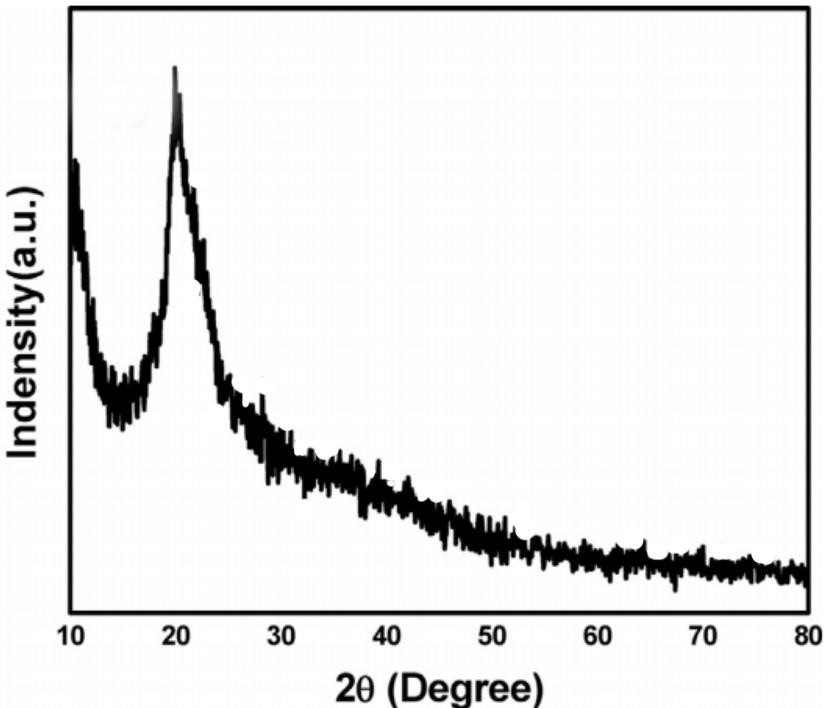


Fig S2B.
